# GRP78-Mediated Signaling Contributes to Axonal Growth Resulting in Motor Function Recovery in Spinal Cord-Injured Mice

**DOI:** 10.3389/fphar.2020.00789

**Published:** 2020-05-29

**Authors:** Yoshitaka Tanie, Tomoharu Kuboyama, Chihiro Tohda

**Affiliations:** ^1^Section of Neuromedical Science, Division of Bioscience, Institute of Natural Medicine, University of Toyama, Toyama, Japan; ^2^Laboratory of Pharmacognosy, Daiichi University of Pharmacy, Fukuoka, Japan

**Keywords:** neuroleukin, axonal growth, 78 kDa glucose regulated protein, spinal cord injury, recovery of motor function

## Abstract

Promoting axonal growth is essential for repairing damaged neuronal connections and motor function in spinal cord injury (SCI). Neuroleukin (NLK) exerts axonal growth activity *in vitro* and *in vivo*, but the mechanism remains unclear. This study reveals that the 78-kDa glucose-regulated protein (GRP78) is a NLK neuronal receptor that contributes to recovery from SCI. Binding and immunoprecipitation assays indicated that NLK binds to GRP78. Pretreatment to cultured neurons with a GRP78-neutralizing antibody suppressed NLK-induced axonal growth. Blocking cell surface GRP78 inhibited neuronal NLK-induced Akt activation. Treatment with an Akt inhibitor suppressed NLK-induced axonal growth. Continuous administration of NLK into the lateral ventricle of SCI mice increased axonal density in the injured region and restored motor function, which was not observed when NLK was simultaneously administered with a GRP78-neutralizing antibody. These results indicate that GRP78 regulates the NLK-induced axonal growth activity; NLK-GRP78 signaling promotes motor function recovery in SCI, presenting as a potential therapeutic target.

## Introduction

Spinal cord injury (SCI) is caused by traumatic damage to the spinal cord and disruption of neuronal relays. The injured neuronal tracts are thought to rarely regrow, resulting in permanent impairment of motor, sensory, and autonomic function. A potentially effective strategy for recovery from these dysfunctions is the restoration of injured neuronal tracts by promoting axonal growth (Courtine and Sofroniew, 2019). After injury, reactive astrocytes form the glial scar surrounding the lesion site and secrete chondroitin sulphate proteoglycans (CSPGs) that inhibit axonal growth (Silver and Miller, 2004; Bradbury and Burnside, 2019). Therefore, overcoming the inhibitory effects of CSPGs on axonal growth is important for accomplishing recovery from motor dysfunction.

Neuroleukin (NLK) has been identified as a cytokine secreted from tumor cells (Liotta et al., 1986). Secreted NLK activates cell motility in an autocrine fashion in tumor cells (Silletti et al., 1991; Watanabe et al., 1991). In addition, NLK has been reported to show neurotrophic factor-like effects on cultured neurons. Previous studies showed that NLK exhibited pro-survival effects in cultured sensory neurons (Gurney et al., 1986) and cultured neural stem cells (Deng et al., 2014). NLK was reported to have an axonal growth activity in the co-culture system of neural stem cells and sertoli cells (Deng et al., 2014). Our previous study also clarified that treatment with recombinant NLK of cultured cortical neurons promoted axonal growth in the absence or presence of CSPGs. A single injection of recombinant NLK to the lesion site increased the axonal density and improved motor function in SCI mice (Tanie et al., 2018). These data reveal that the axonal growth activity of NLK may be beneficial for functional recovery in SCI. However, the signaling pathway of NLK-induced axonal growth has not been elucidated. In the current study, we found a novel neuronal receptor of NLK, the 78-kDa glucose-regulated protein (GRP78). Furthermore, NLK-GRP78 signaling was shown to be pivotal for axonal growth and functional recovery in SCI mice.

## Materials and Methods

All experiments were performed in accordance with the Guidelines for the Care and Use of Laboratory Animals of the University of Toyama, Japan. The Committee for Animal Care and Use at the Sugitani Campus of the University of Toyama approved the study protocols. The approval number for the animal experiments was A2016INM-3. All efforts were made to minimize the number of animals used.

### Primary Neuronal Culture

Primary cultured cerebral cortical neuronal cells were prepared from ddY mice (Japan SLC, Hamamatsu, Japan) at embryonic day 14 (E14) as described previously (Tohda et al., 2012). Eight-well chamber slides coated with 5 µg/ml poly-D-lysine (PDL; Sigma-Aldrich, St. Louis, MD, USA) were used for cultures. The cells were cultured on PDL-coated slides with neurobasal medium (Life Technologies, Carlsbad, CA, USA) containing 12% horse serum (Life Technologies), 0.6% glucose, 2 mM L-glutamine (HS medium) at 37° in a humidified incubator with 10% CO_2_. Four hours later, the medium was replaced with fresh neurobasal medium containing 2% B-27 supplement (Life Technologies) without horse serum (B27 medium).

### Axonal Growth Assay

Cultured cortical neurons were treated with functional blocking antibodies against the autocrine motility factor receptor (AMFR; 1, 10, and 100 ng/ml, Cat. No. NBP2-15734, Novus Biologicals, Littleton, CO, USA), 78-kDa glucose regulated protein (GRP78; 10, 100 ng/ml, Cat. No. ab108613, Abcam, Cambridge, UK), or normal rabbit immunoglobulin (IgG; 1, 10, and 100 ng/ml, Cat. No. sc-2027, Santa Cruz Biotechnology, Dallas, TX, USA) at 1 day after culture started. In our preliminary experiments, when cultured neurons were treated with 2 µg/ml neutralizing antibody for AMFR, according to a previous report (Lucarelli et al., 2015), antibody cell toxicity was observed. Therefore, the concentration of the neutralizing antibodies used was based on the maximum concentration (100 ng/ml) with no cell toxicity (data not shown). In the case of the antibody against GRP78, the maximum antibody concentration (100 ng/ml) that did not produce cell toxicity was used. After 15 min of treatment with the neutralizing antibodies, 100 ng/ml recombinant NLK (His-tagged, Cat. No. ATGP0348, ATGen, Seongnam, Korea) or vehicle (sterile distilled water) were administered to the cells and incubated for 5 days. For Akt inhibition, cortical neurons were cultured for 1 day *in vitro*, and then treated with 100 ng/ml recombinant NLK and/or 0.1 µM Akt inhibitor (Tokyo Chemical Industry Co., Ltd.) for 5 days. Five days post treatment, the cells were fixed with 4% paraformaldehyde at room temperature (RT) and immunostained with a mouse anti-phosphorylated neurofilament-H (pNF-H) monoclonal antibody (1:300; Cat. No. SMI-35R, Covance, Emeryville, CA, USA), an axonal marker, and a rabbit anti-microtubule-associated protein 2 (MAP2) polyclonal antibody (1:2,000; Cat. No. ab32454, Abcam), a neuronal marker at 4°. Alexa Fluor 594-conjugated goat anti-mouse IgG (1:400; Cat. No. A-11005, Life Technologies) and Alexa Fluor 488-conjugated goat anti-rabbit IgG (1:400; Cat. No. A-11008, Life Technologies) were used as secondary antibodies, respectively in second antibody reaction at RT. Nuclei were counterstained with 1 µg/ml 4′,6-diamidino-2-phenylindole (DAPI; Enzo Life Science, Farmingdale, NY, United states). Fluorescent images were captured using a Cell Observer Z1 fluorescent microscope (Call Zeiss) at a photo size of 432.49 µm x 322.81 µm. The lengths of pNF-H-positive axons were measured using MetaMorph version 7.8 (Molecular Devices). The sum of the axon lengths was divided by the number of MAP2-positive neurons in each photo to calculate the mean axonal length.

### Drug Affinity Responsive Target Stability Analysis

Lysates of the plasma membrane fraction of cultured neurons were prepared with the Mem-PER Plus Membrane Protein Extraction Kit (Thermo Fisher Scientific, Waltham, MA, USA) after 6 days of *in vitro* culturing following the manufacturer’s protocol. The protein concentration of the cell lysates was measured using a Pierce 660 nm Protein Assay Kit (Thermo Fisher Scientific). Drug affinity responsive target stability (DARTS) analysis was performed as described previously (Tohda, C., Urano, T., Umezaki, M., Nemere, I., Kuboyama, T., 2012). Cell lysates of cultured neurons containing 5 µg protein were incubated for 30 min at RT with 10 µg/ml recombinant NLK or vehicle. Thereafter, the mixture was proteolyzed with thermolysin (Wako) in reaction buffer containing 50 mM Tris–HCl, pH 8.0; 50 mM NaCl; 10 mM CaCl_2_ for 30 min at 37°C (thermolysin: protein, 0.0005 µg: 5 µg). At the end of the reaction period, 0.5 M ethylenediaminetetraacetic acid (EDTA), pH 8.0 was added to each sample at a 1:10 ratio on ice to stop proteolysis. Samples were incubated with NuPAGE lithium dodecyl sulphate (LDS) sample buffer (Life Technologies, Carlsbad, CA, USA) and 5% 2-mercaptoethanol at 75° for 5 min. The samples were loaded onto 8% polyacrylamide gels and electrophoresed. After electrophoresis, proteins in the gel were transferred to a nitrocellulose membrane (Bio-Rad, Berkeley, CA, USA) and blocked with 0.1% Tween 20 in Tris buffered saline (T-TBS) containing 5% skim milk (Wako) at RT. Subsequently, the membrane was gently washed with T-TBS and incubated with a goat anti-GRP78 polyclonal antibody (1:1,000; Cat. No. sc-1050, Santa Cruz) in Can Get Signal solution 1 (Toyobo, Osaka, Japan) overnight at 4°C. After washing with T-TBS, the membrane was incubated with a donkey anti-goat IgG horse-radish peroxidase (HRP)-conjugated secondary antibody (1:5,000; Cat. No. sc-2020, Santa Cruz) in Can Get Signal solution 2 (Toyobo) for 2 h at RT. After washing, the antibody signal was detected with enhanced chemiluminescence (ECL) Prime Western Blotting Detection Reagent (GE Healthcare, Buckinghamshire, UK) using an ImageQuant LAS 4000 system (GE Healthcare). The signal intensities were quantified using a CS analyzer (ATTO, Tokyo, Japan).

### Binding Assay by Immunoprecipitation

Twenty pmol recombinant NLK and 10 pmol recombinant GRP78 (Cat. No. SPR-119A, Stress Marq Biosciences Inc., Victoria, British Columbia, Canada) were coincubated for 1 h at RT. Fifty µl of Dynabeads Protein G (Life Technologies) were treated with 1% bovine serum albumin (BSA) for blocking in 0.1% Tween phosphate buffered saline (T-PBS) for 30 min at 4° with rotation. Then, the protein G was incubated with a mouse anti-His-tag monoclonal antibody (1 µg, Cat. No. LS-C51081, Life Span Biosciences Inc. Seattle, WA, US) or normal mouse IgG (1 µg, Cat. No. sc-2025, Santa Cruz) for 30 min at 4° with rotation. To crosslink protein G with antibodies, the complex of protein G and antibodies was incubated in 50 mM dimethyl pimelimidate dihydrochloride (Tokyo Chemical Industry Co., Ltd., Tokyo, Japan) for 1 h at RT with rotation. After washing protein G, incubated NLK and GRP78 were added to the washed protein G and the mixture was incubated for 2 h at RT with rotation. For the elution of immunoprecipitants, the samples were mixed with LDS sample buffer and 0.1 M glycine-HCl (pH 2.8) for 5 min at 95° and then were loaded onto 8% polyacrylamide gels and electrophoresed. After electrophoresis, proteins in the gel were transferred to a nitrocellulose membrane (Bio-Rad, Berkeley, CA, USA) and blocked with 0.1% T-TBS containing 5% skim milk at RT. Subsequently, the membrane was gently washed with T-TBS and incubated with a rabbit anti-GRP78 monoclonal antibody (1:2,000; Abcam) in Can Get Signal solution 1 overnight at 4°C. After washing with T-TBS, the membrane was incubated with a goat anti-rabbit IgG HRP-conjugated secondary antibody (1:2,000; Cat. No. sc-2004, Santa Cruz Biotechnology) in Can Get Signal solution 2 for 2 h at RT. After washing, antibodies were detected with chemiluminescence as described above. Antibodies on the membrane were then stripped with Western blot stripping solution (Nacalai Tesque, Kyoto, Japan) and the membrane was incubated with a mouse anti-NLK monoclonal antibody (1:1,000; Cat. No. ab66340, Abcam) in Can Get Signal solution 1 overnight at 4°C. After washing with T-TBS, the membrane was incubated with a goat anti-mouse IgG HRP-conjugated secondary antibody (1:10,000; Cat. No. 97040, abcam) in Can Get Signal solution 2 for 2 h at RT. Antibodies were detected with chemiluminescence as described above.

### Detection of Akt Phosphorylation in Neuronal Lysates

At 6 days *in vitro*, cultured cortical neurons were pretreated with the neutralizing antibody for GRP78 (100 ng/ml, Abcam) or normal rabbit IgG (100 ng/ml, Santa Cruz Biotechnology) for 15 min. Thereafter, 100 ng/ml NLK or vehicle solutions were added to the cells for 30 min. For the preparation of cell lysates, the cells were washed with PBS and then incubated for 20 min on ice with mammalian protein extraction reagent (M-PER) lysis buffer (Thermo Fisher Scientific) containing a protease and phosphate inhibitor cocktail (Thermo Fisher Scientific). After incubation, the cell solution was centrifuged (14,000 × g, 10 min, 4°) to remove the cell debris, and the supernatants were used as cell lysates. The cell lysate protein concentration was measured as mentioned above. For detecting phosphoserine proteins in the lysate, a similar procedure of immunoprecipitation as in the binding assay was performed. The neuronal lysate containing 50 µg protein and rabbit anti-phosphoserine polyclonal antibody (0.5 µg, Cat. No. AB1603, Merck Millipore, Burlington, MA, united states) or normal rabbit IgG (0.5 µg, Cat. No. sc-2027, Santa Cruz Biotechnology) were used in this experiment. Western blotting was performed using a rabbit anti-Akt polyclonal antibody (1:1,000; Cat. No. 9272, Cell Signaling Technology, MA, United States) and goat anti-rabbit IgG HRP-conjugated secondary antibody (1:2,000; Cat. No. sc-2004, Santa Cruz Biotechnology).

### Continuous Administration of NLK to SCI Mice

All mice were housed with ad libitum access to food and water and kept in a constant environment (22 ± 2°C, 50 ± 5% humidity, 12 h light cycle starting at 07:00). Eight-week-old female ddY mice (SLC, Japan) were used for SCI experiments. The mice were anesthetized with butorphanol tartrate (5 mg/kg, i.p., Meiji Seika Pharma Co., Ltd., Tokyo, Japan), medetomidine hydrochloride (0.75 mg/kg, i.p., Zenyaku Kogyo Co., Ltd., Tokyo, Japan) and midazolam (4 mg/kg, i.p., Fuji Pharma Co., Ltd., Tokyo, Japan). After laminectomy, contusion injury was established by dropping a 6.5-g weight from a height of 3 cm onto the exposed spinal cord at the level of T11–T12 using a stereotaxic instrument (Narishige, Tokyo, Japan), as described previously (Shigyo and Tohda, 2016).

**Figure 1:**
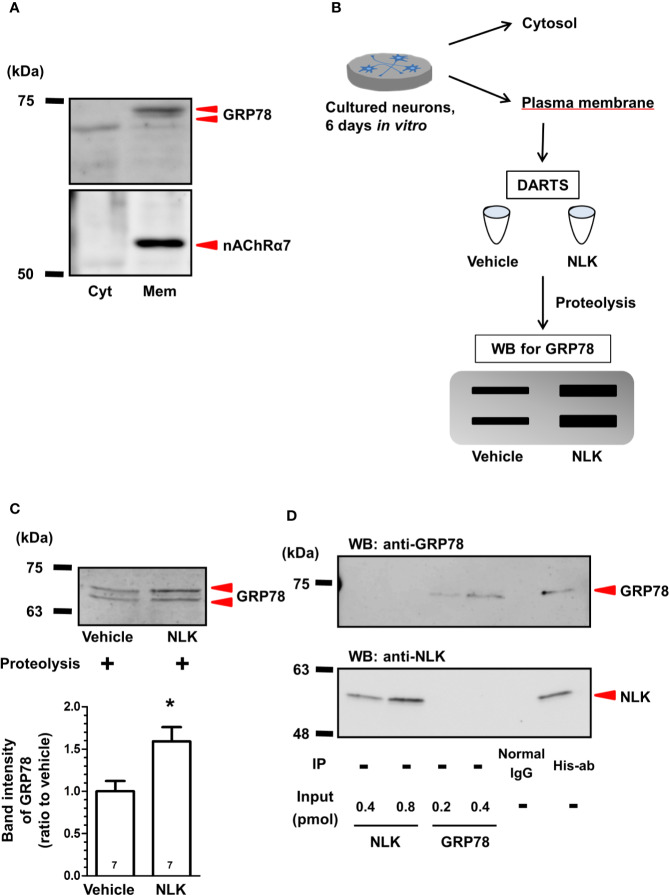
Neuroleukin (NLK) binds to 78-kDa glucose-regulated protein (GRP78) in cultured neurons. **(A)** In plasma membrane lysates of cultured neurons, GRP78 and nicotinic acetylcholine receptor α7 (nAChRα7) were detected by Western blot. **(B)** After drug affinity responsive stability reaction, GRP78 in the membrane-associated lysates were detected using Western blot. **(C)** Accumulated intensities of GRP78 in the lysates were quantified in relation to expression of the vehicle-treated cultures. *p < 0.05 vs Vehicle, unpaired two-tailed *t*-test. The numbers in the graph bars indicate the number of experiments. **(D)** Recombinant NLK (20 pmol) and recombinant GRP78 (10 pmol) were co-incubated and were immunoprecipitated using a His-tag antibody or normal mouse IgG. NLK and GRP78 in the immunoprecipitant were detected by Western blot. Only recombinant NLK (0.4, 0.8 pmol) and recombinant GRP78 (0.2, 0.4 pmol) were electrophoresed as input.

After SCI surgery, the mice were placed in a stereotaxic apparatus and the head was kept in a fixed position. The scalp was shaved, followed by a sagittal midline incision to expose the skull. A cannula (Brain Infusion Kit 3, Alzet, Cupertino, CA, United states) was inserted to a lateral ventricle as follows: bregma −0.22 mm, lateral to the left +1 mm and −2.5 mm depth. The free end of the cannula was connected to a micro-osmotic pump (Alzet model 1004) *via* a 3.5 cm piece of polyvinylchloride (PVC) tubing (Alzet). The pump was placed into a subcutaneous pocket on the back of the mouse. The cannula was fixed to the skull with Aron Alpha A “Sankyo” (Daiichi Sankyo, Tokyo, Japan). The infusion rate of the micro-osmotic pump was 0.11 μl/hr. The filled pumps were incubated in 0.9% sterile saline at 37°C for at least 16 hr in a CO_2_ incubator before implantation. As the vehicle solution, artificial cerebrospinal fluid (ACSF) (containing 130 mM NaCl, 24 mM NaHCO_3_, 3.5 mM KCl, 1.3 mM NaH_2_PO_4_, 2 mM CaCl_2_, 2 mM MgCl_2_·6H_2_O, and 10 mM glucose at pH 7.4) was filled into the micro-osmotic pump and connected PVC tube. The micro-osmotic pump and tube were filled with 100 μg/ml of NLK (ATGen), 100 μg/ml GRP78 (Abcam) antibody or 100 μg/ml normal IgG (Santa Cruz Biotechnology) that was dissolved in ACSF considering that the pump efflux was 0.11 µl/hr and CSF was produced at a speed of 0.325 µl/min (Casaca-Carreira et al., 2018). Thus, the final concentrations of NLK and antibodies were always approximately 65 ng/ml when they were delivered to the CSF of SCI mice. These doses are enough for inducing axonal growth by NLK and inhibiting the effect of NLK by the antibodies (**Figure 1**). During and after surgery, the mice were placed on a heating pad to maintain body temperature. Basso mouse scale (BMS) (Basso et al., 2006) and Toyama mouse score (TMS) (Shigyo et al., 2014) were used to evaluate hindlimb motor function of the SCI mice in an open field (black color, 50.0 × 42.5 × 15.0 cm) under 500-luxillumination. The behavioral evaluation started at 1 day post injury and was performed once a day for 21 days. The number of used animals was listed as follows.

**Table:** 

Group	Number of animals
Normal IgG/ Vehicle	8
Normal IgG/ NLK	8
GRP78-ab/ NLK	9

### Immunohistochemistry

At day 21 post injury, the mice were anesthetized and perfusion-fixed with 4% paraformaldehyde (PFA) in PBS. The spinal cord tissues, including injured regions, were immersed in 30% sucrose solution. Spinal cords were sagittally sectioned at a thickness of 14 µm using a CM1860UV cryostat (Leica, Heidelberg, Germany). The sections were immunostained with a chicken anti-NF-H IgY polyclonal antibody (1:1,000; Cat. No. AB5539, Chemicon, Temecula, CA, USA). Alexa Fluor 488-conjugated goat anti-chicken IgY (1:400; Cat. No. A-11039, Life Technologies) was used as a secondary antibody. To decide the lesion area, nuclei were counterstained with 1 µg/ml DAPI (Enzo Life Science). Fluorescence images were obtained using an inverted microscope system (BZ-X710; Keyence, Osaka, Japan) with a 20× NA 0.75 objective lens (CFI PlanApo-λ; Nikon Instech, Tokyo, Japan). The injured regions were defined by the areas that accumulated DAPI-positive nuclei. The area of the injured region and the length of NF-H-positive axons were measured using MetaMorph version 7.8 (Molecular Devices).

### Statistical Analysis

Statistical comparisons were performed with a one-way analysis of variance (ANOVA) with the *post hoc* Bonferroni test, repeated measures two-way ANOVA with *post hoc* Bonferroni test, split plot ANOVA *post hoc* Tukey’s HSD test, or an unpaired two-tailed t-test using GraphPad Prism 5 software (GraphPad Software, San Diego, California, USA) or JMP 14 (SAS institute, Cary, NC, United states). P < 0.05 was considered statistically significant. The data are presented as the mean ± SE.

**Table:** 

Figure	Study	Analysis
1C	Binding assay	Unpaired two-tailed *t-*test.
2, 3, 4C	Axonal growth assay	One-way ANOVA *post hoc* Bonferroni’s multiple comparison test.
4B	Phosphorylation assay	One-way ANOVA *post hoc* Bonferroni’s multiple comparison test.
5A, 5B	In vivo experiments	Repeated measures using two-way ANOVA. *Post hoc* Bonferroni test.
5E		Split plot ANOVA *post* hoc Tukey’s HSD test.
5F		One-way ANOVA.

## Results

### Direct Binding of NLK to GRP78

Our previous study indicated that the cell surface GRP78 is a receptor for NLK in astrocytes (Tanie et al., 2018). Therefore, we hypothesized that this molecule may also function as a receptor of NLK in neurons. Although the role of GRP78 as a cytosolic protein that belongs to the heat-shock protein family has been studied thoroughly (Casas, 2017), few studies have reported about the functions of GRP78 as a signal transduction receptor in cultured neurons (Goldenberg-Cohen et al., 2012; Bellani et al., 2014; Louessard et al., 2017). Therefore, we tested that GRP78 was, indeed, expressed at the cell surface of cultured neurons. Plasma membrane-derived lysates and cytosolic lysates were prepared and examined by Western blot. The α7 nicotinic acetylcholine receptor (nAChRα7) was used as a marker of a plasma membrane-specific protein and was detected exclusively in the plasma membrane-derived lysate but not in the cytoplasm one (**Figure 1A**), confirming appropriate separation of plasma membrane from cytosolic protein fractions. As expected, GRP78 was detected in both cytosolic and plasma membrane-derived lysates (**Figure 1A**).

To examine the direct binding of NLK to cell surface GRP78 in neurons, DARTS analysis (Lomenick et al., 2009) was used (**Figure 1B**). In this method, when a ligand binds a target protein, the structural conformation of the protein is modified. As a result, resistance against proteolysis is changed, and it becomes harder or easier to degrade the target protein (Lomenick et al., 2009). In the current study, plasma membrane lysates of cultured neurons were incubated with recombinant NLK or vehicle. After the proteolysis reaction using thermolysin, the lysates were electrophoresed and GRP78 was detected by Western blot. Accumulated intensity of GRP78 was normalized to that of the vehicle-treated samples and was found significantly increased by NLK treatment (**Figure 1C**). This result suggests that NLK interacts with GRP78 at the neuronal plasma membrane and this binding increases the resistance of GRP78 against proteolysis. We then examined whether NLK interacts with GRP78 by direct binding using immunoprecipitation. Protein samples immunoprecipitated with an anti-His-tag antibody revealed expression of both NLK and GRP78, while no bands were detected in samples immunoprecipitated with normal IgG (**Figure 1D**), indicating that NLK directly interacts with GRP78. Doublet bands of GRP78 were detected in the case of using cell lysate (**Figures 1A, C**) but not the recombinant protein (**Figure 1D**). From these results, part of lysate-derived GRP78 might be subjected to post-translational modifications.

### GRP78 Involved in NLK-Induced Axonal Growth

**Figure 2:**
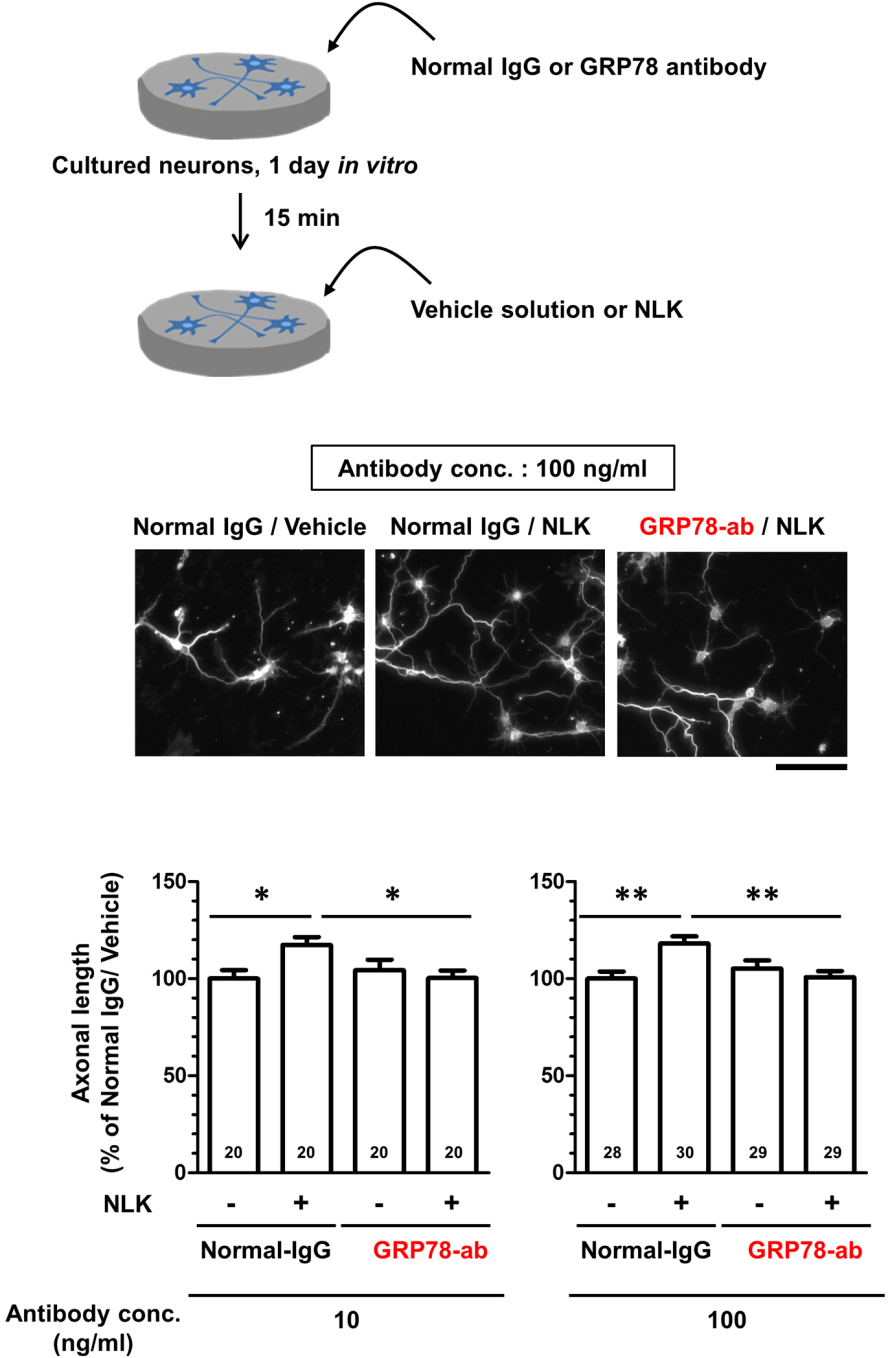
78-kDa glucose-regulated protein (GRP78) participates in neuroleukin (NLK)-induced axonal growth. At 1 day *in vitro*, cultured cortical neurons were treated with GRP78 neutralizing antibody (GRP78-ab, 10 or 100 ng/ml) or normal IgG (10 or 100 ng/ml) for 15 min. Subsequently, the cells were treated with NLK (100 ng/ml) or Vehicle solution. Five days after the treatment, the cells were fixed and double immunostained for phosphorylated neurofilament-H (pNF-H) and microtubule-associated protein 2 (MAP2). Representative images of pNF-H-positive axons are shown. Scale bar = 100 µm. Total lengths of pNF-H-positive axons per neuron were quantified for each treatment. *p < 0.05, **p < 0.01, one-way ANOVA *post hoc* Bonferroni’s multiple comparison test. Numbers in columns show the number of captured images.

As shown in **Figure 1**, GRP78 was expressed at the plasma membrane of neurons and bound to NLK. To investigate whether NLK promotes axonal growth *via* GRP78, a neutralizing antibody against GRP78 was used for blocking cell surface GRP78 expression. In the normal IgG-treated group, treatment with recombinant NLK significantly increased the axonal density in the cultured cortical neurons. In contrast, the neutralizing antibody against GRP78 diminished NLK-induced axonal growth (**Figure 2**). Normal IgG and the neutralizing antibody against GRP78 were used at two doses (10 and 100 ng/ml) and the same results were observed in both cases. These results indicate that the cell-surface GRP78 is necessary for NLK-mediated axonal growth in neurons.

### Autocrine Motility Factor Receptor Is Not Involved in the Axonal Growth Activity of NLK

**Figure 3:**
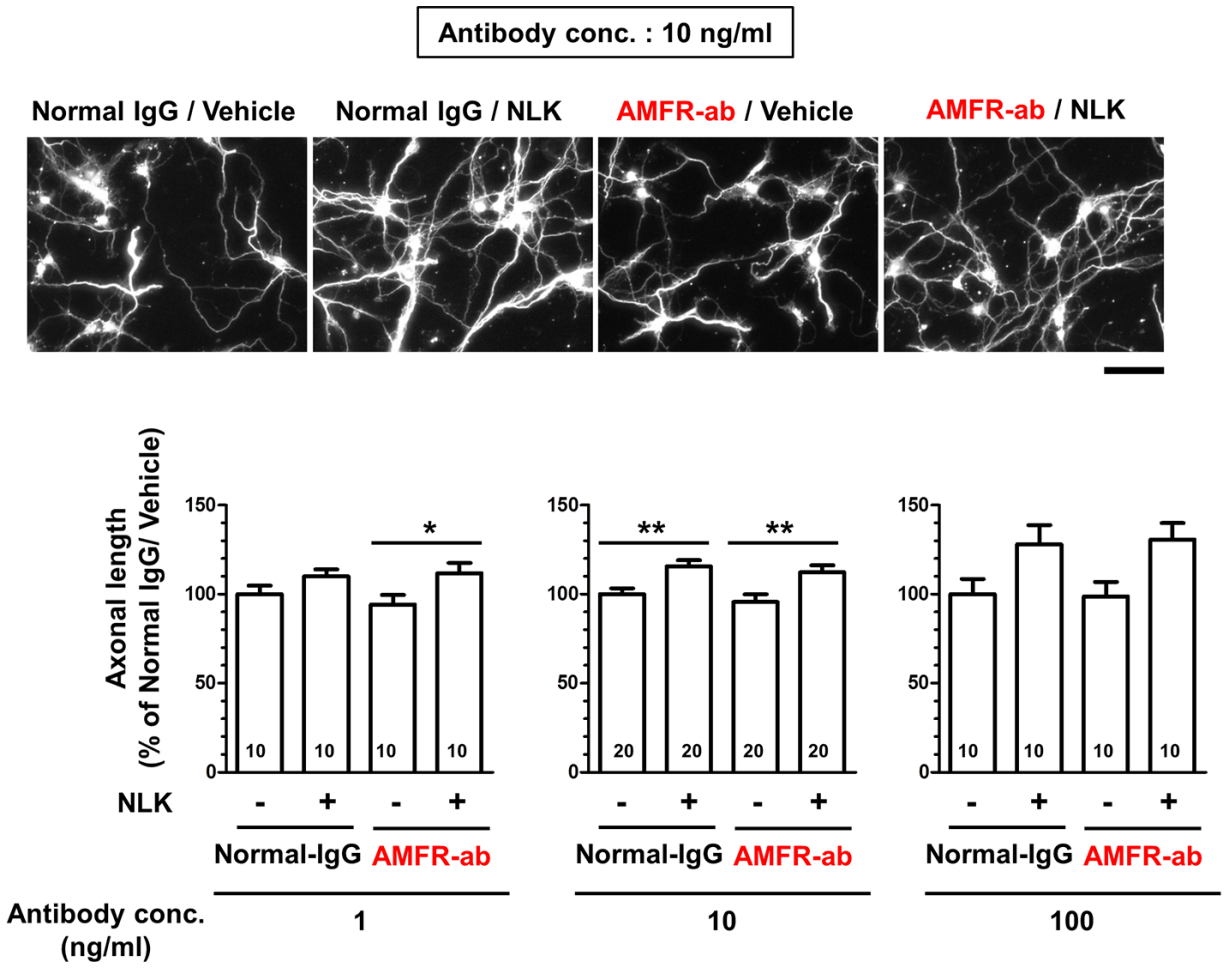
Autocrine motility factor receptor (AMFR) is not involved in the axonal growth activity of neuroleukin (NLK). At 1 day *in vitro*, a neutralizing antibody for AMFR (AMFR-ab, 1, 10, and 100 ng/ml) or normal IgG (1, 10, and 100 ng/ml) was added to cultured cortical neurons for 15 min. After 15 min, the cells were treated with NLK (100 ng/ml) or vehicle solution for 5 days, and then fixed and double immunostained for phosphorylated neurofilament-H (pNF-H) and microtubule-associated protein 2 (MAP2). Representative images of pNF-H-positive axons are shown. Scale bar = 100 µm. *p < 0.05, **p < 0.01, one-way ANOVA *post hoc* Bonferroni’s multiple comparison test. Numbers in columns show the number of captured images.

Autocrine motility factor receptor (AMFR) is reported as a receptor for NLK. This receptor is a seven transmembrane-type receptor that promotes cell motility *via* NLK stimulation in tumor cells (Silletti et al., 1991; Shimizu et al., 1999; Fairbank et al., 2009). A previous study has shown that AMFR is expressed in cultured neurons (Leclerc et al., 2000). It has also been reported that NLK recognizes an N-glycosylation site of AMFR located at its C-terminal region and other extracellular domain located at C-terminal of AMFR (Haga et al., 2006). In this study, we used a neutralizing antibody that binds to the C-terminal region of AMFR to inhibit the interaction of NLK with AMFR. Primary cultured cortical neurons were treated with NLK and the neutralizing antibody at 1 day *in vitro* culture. Five days after treatment, the axonal length tended to increase by NLK stimulation in both the normal IgG-treated groups and neutralizing antibody-treated groups with each concentration of antibodies (**Figure 3**). Normal IgG and the neutralizing antibody for AMFR were used at three doses (1, 10, and 100 ng/ml), which resulted in similar trends. These results showed that AMFR masking had no effect on NLK-induced axonal growth, indicating that AMFR is not involved in the axonal growth activity of NLK.

### NLK Accelerates Akt Signaling in Neurons Through Cell Surface GRP78

**Figure 4:**
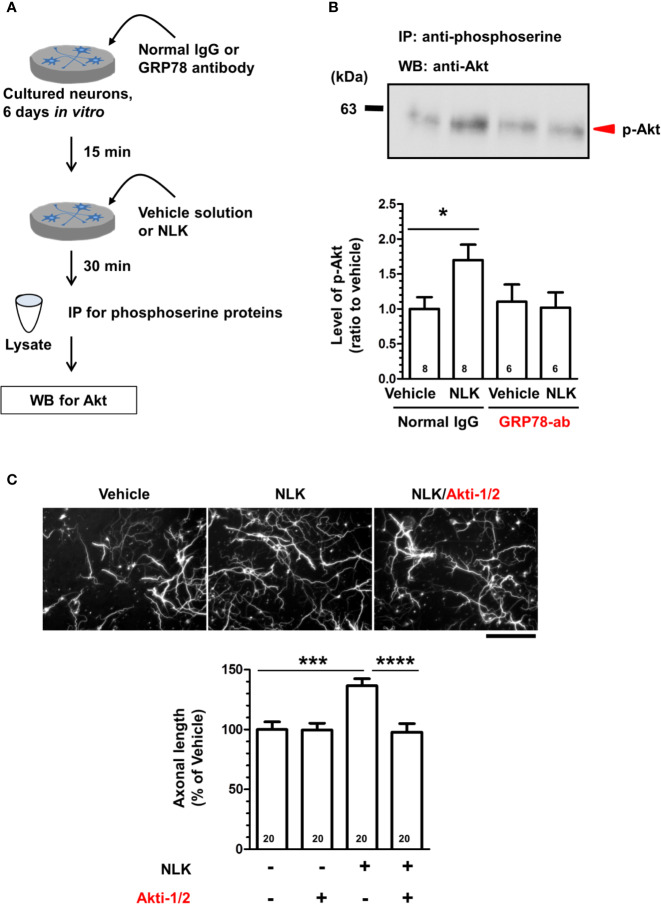
Neuroleukin (NLK) accelerates Akt signaling *via* the 78-kDa glucose-regulated protein (GRP78), resulting in promotion of axonal growth. **(A)** Cortical neurons were cultured for 6 days, and then treated with a GRP78 antibody (GRP78-ab, 100 ng/ml) or normal IgG (100 ng/ml) for 15 min. Thereafter, NLK (100 ng/ml) was administered to the cells. At 30 min post NLK-treatment *in vitro*, cell lysates were recovered, and serine phosphorylated-proteins in the lysates were immunoprecipitated using phosphoserine antibody. The level of Akt in the phosphorylated proteins was detected by Western blot. **(B)** The band intensity of phosphorylated Akt was quantified. *p < 0.05, one-way ANOVA *post hoc* Bonferroni’s multiple comparison test. Numbers in columns show the number of experiments. **(C)** After 1 day of *in vitro* culture, NLK (100 ng/ml) and/or Akt inhibitor (Akt-1/2, 0.1 µM) were simultaneously added to cultured cortical neurons for 5 days. The cells were fixed and double immunostained for phosphorylated neurofilament-H (pNF-H) and microtubule-associated protein 2 (MAP2). Representative images of pNF-H-positive axons are shown. Scale bar = 100 µm. Total lengths of pNF-H-positive axons per neuron were quantified for each treatment. ***p < 0.001, ****p < 0.0001, one-way ANOVA *post hoc* Bonferroni’s multiple comparison test. Numbers in columns show the number of captured images.

In tumor cells and endothelial cells, stimulation with ligands of cell surface GRP78, such as α2-macroglobulin, T-cadherin, Cripto, and insulin-like growth factor 1 receptor, induces activation of the PI3K-Akt signaling (Misra et al., 2006; Philippova et al., 2008; Kelber et al., 2009; Yin et al., 2017). In the plasma membrane, GRP78 interacts with PI3K, an activator of Akt, leading to the production of PIP_3_ (Liu et al., 2013; Zhang et al., 2013). In addition, treatment to cultured cortical neurons with recombinant NLK also induces Akt activation (Deng et al., 2014); however, the plasma membrane proteins involved in this process remain unknown. Therefore, we predicted that the cell surface GRP78 was involved in inducing Akt activation and axonal growth by NLK in neurons. Primary cultured cortical neurons were treated with NLK and a neutralizing antibody for GRP78 or normal IgG at 6 days *in vitro* culture. Thirty minutes after the treatment, cell lysates were prepared, phosphorylated-Akt proteins were immunoprecipitated with a phospho-serine antibody and detected by Western blot (**Figure 4A**). As a result, the level of phosphorylated-Akt was significantly elevated by NLK treatment in the normal IgG-treated group. However, NLK-induced phosphorylation of Akt was diminished by treatment with a neutralizing antibody against GRP78 (**Figure 4B**). This result suggests that NLK-induced Akt activation is mediated by cell surface GRP78 in neurons. Next, we investigated whether Akt activation is needed for NLK-induced axonal growth. Cortical neurons were cultured for 1 day and then treated with NLK and Akt inhibitor for 5 days. Treatment with the Akt inhibitor completely diminished the axonal growth activity of NLK (**Figure 4C**). These results indicate that Akt is a downstream molecule of the NLK-GRP78 signaling that promotes axonal growth.

### NLK-GRP78 Signaling Increases Axonal Density and Improves Motor Function in Spinal Cord-Injured Mice

**Figure 5:**
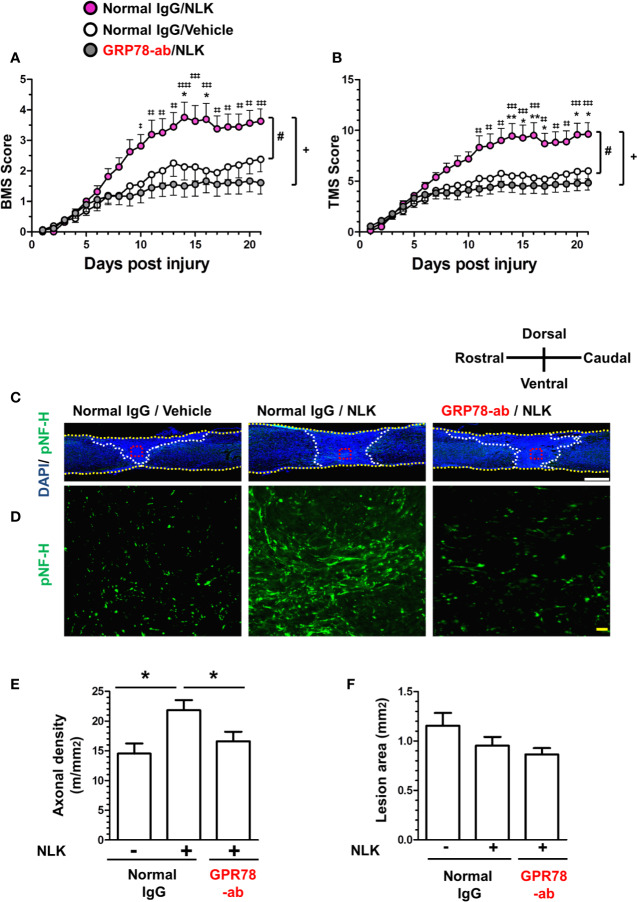
GRP78 is involved in the NLK-mediated axonal growth activity and recovery of motor function in the spinal cord injury (SCI) mouse. Normal IgG/NLK (pink circles, 8 mice, 16 hindlimbs, n = 16) or Normal IgG/Vehicle (white circles, 8 mice, n = 16) or GRP78 antibody/NLK (gray circles, 9 mice, n = 18) were continuously administered to the lateral ventricle of SCI mice at the day post injury. Hindlimb motor function of SCI mice was assessed using the Basso mouse scale [BMS **(A)**] and the Toyama mouse score [TMS **(B)**]. ^#^p < 0.05 vs Normal IgG/Vehicle, ^+^p < 0.05 vs GRP78-ab/NLK, drug × day interaction by repeated measures using two-way ANOVA. *p < 0.05, **p < 0.01 vs Normal IgG/Vehicle, ^‡^*p* < 0.05, ^‡‡^*p* < 0.01, ^‡‡‡^*p* < 0.001, ^‡‡‡‡^*p* < 0.0001 vs GRP78-ab/NLK at each time point, *post hoc* Bonferroni test. At 21 days post injury, spinal cord tissues were prepared, and sagittal sections of spinal cords were immunostained for phosphorylated neurofilament-H (pNF-H). Nuclei were counterstained using 4′,6-diamidino-2-phenylindole (DAPI). Figure 5C shows representative immunofluorescence merge images of DAPI and pNF-H. Regions enclosed with red dot lines in figure 5C are magnified in Figure 5D, showing images of pNF-H-positive axons. The yellow dot lines indicate the outline of the spinal cords. The white dot lines show the border of the lesion area. The axonal density of pNF-H-positive axons at the lesion site **(E)** and the size of the injured region **(F)** were quantified. *p < 0.05, split plot ANOVA *post hoc* Tukey’s HSD test. White scale bar = 500 μm **(C)**, Blue scale bar = 20 μm **(D)**.

In SCI, disruption of the neuronal relay occurs resulting in enduring motor dysfunction. After injury, reactive astrocytes form glial scars surrounding the lesion site and secrete CSPGs, leading to inhibition of axonal regrowth and recovery of motor function (Silver and Miller, 2004; Bradbury and Burnside, 2019). Our previous study showed that treatment to cultured neurons with NLK promoted axonal growth in the presence of CSPGs. The single injection of NLK to the lesion site promoted axonal growth and improved motor function in SCI mice (Tanie et al., 2018). In addition, as shown in **Figures 3** and **4**, cell surface GRP78 was necessary for Akt activation and NLK-induced axonal growth. To investigate the effect of NLK-GRP78 signaling on axonal growth *in vivo*, SCI mice were used. After injury, continuous administration of NLK or vehicle solution (ACSF) into the lateral ventricle was immediately performed using micro-osmotic pump for 21 days. The concentrations of NLK and antibodies were maintained at approximately 65 ng/ml in the CSF during the administration period. Our previous study demonstrated that 10–100 ng/ml NLK promoted axonal growth in cultured neurons (Tanie et al., 2018). In addition, pretreatment with 10–100 ng/ml neutralizing antibody against GRP78 diminished the axonal growth activity of NLK (**Figure 1**). We also confirmed that 10–100 ng/ml NLK had no effect in cultured astrocytes (Tanie et al., 2018). A neutralizing antibody against GRP78 (approximately 65 ng/ml in CSF) or normal IgG was simultaneously administered with NLK (approximately 65 ng/ml in CSF) or vehicle solution. The hindlimb motor functions were evaluated by BMS (**Figure 5A**) and TMS (**Figure 5B**). The scores of normal IgG/NLK-treated mice were significantly elevated compared with the scores of normal IgG/vehicle-treated mice for 21 days after injury. In each scoring, two-way repeated measures ANOVA showed a significant difference for time×drug interactions among the groups ([normal IgG/vehicle vs. normal IgG/NLK] F(20,600) = 3.74, *p* < 0.0001 in BMS; (20,600) = 4.38, *p* < 0.0001 in TMS; [normal IgG/NLK vs. GRP78-ab/NLK] F(20,640) = 9.20, *p* < 0.0001 in BMS; F(20,640) = 8.82, *p* < 0.0001 in TMS). During the 11–21 days post injury, *post hoc* Bonferroni analysis indicated that both scores of the GRP78 antibody-treated groups were significantly decreased compared to those of the NLK-treated groups (**Figures 5A, B**).

At 21 days after injury, spinal cord tissues were isolated, and slices of spinal cord that included the lesion area were prepared for immunohistochemistry and immunostained with the axonal marker NF-H (**Figures 5C, D**). To decide the lesion area, DAPI was used for nuclear counterstaining (**Figure 5C**). In the lesion site, the density of NF-H-positive axons was significantly increased by NLK treatment, compared with vehicle-treated groups. In addition, administration of a neutralizing antibody for GRP78 significantly attenuated the increase in axonal density by NLK administration in the lesion site (**Figure 5E**). In this experiment, the size of lesion was not changed by treatments (**Figure 5F**). These results suggest that GRP78 has important roles in the axonal growth activity of NLK and the recovery of SCI mice from motor dysfunction.

## Discussion

Although previous reports have shown that NLK has an axonal growth activity *in vitro* and *in vivo* (Deng et al., 2014; Tanie et al., 2018), the mechanisms of these actions have not been elucidated yet. The present study indicates that the cell surface-expressed GRP78 is a NLK receptor that regulates axonal growth signaling. Our study revealed NLK-GRP78 signaling as a new mechanism for the promotion of axonal growth and Akt was identified as a downstream molecule. Several studies have reported that Akt activation is induced by ligand stimulation of cell-surface GRP78 and is necessary for regulating cellular functions, such as cell survival, motility, and proliferation, in tumor cells and endothelial cells (Misra et al., 2006; Philippova et al., 2008; Kelber et al., 2009; Yin et al., 2017). Here, we show an additional function of Akt activation: its involvement in axonal growth activity mediated by the cell surface GRP78. Furthermore, this study demonstrates that NLK-GRP78 signaling promotes axonal growth at the lesion site and enhances recovery of motor function in SCI mice.

GRP78 belongs to the heat-shock protein family and works as a chaperone protein as well as a signal transduction receptor for several ligands at the plasma membrane (Gonzalez-Gronow et al., 2009; Ni et al., 2011). A few studies have reported the downstream signaling of cell surface GRP78 in neurons (Casas, 2017). For example, binding of tissue-type plasminogen activator to GRP78 suppressed the deleterious activation of the PERK branch pathway, leading to neuroprotection in the brain of a mouse ischemia model (Louessard et al., 2017). Several studies have reported the PI3K-Akt signaling as one of the downstream pathways of cell surface GRP78 (Misra et al., 2006; Philippova et al., 2008; Kelber et al., 2009; Yin et al., 2017). Binding of GRP78 to PI3K is required for production of PIP_3_, which is involved in Akt activation at the plasma membrane in tumor cells (Liu et al., 2013; Zhang et al., 2013). PI3K-Akt signaling is also involved in neurite outgrowth (Read and Gorman, 2009). The results emerging from the present study indicate that Akt signaling is also related to NLK-induced axonal growth *via* cell surface GRP78 in neurons (**Figure 4**). We have previously reported that cell surface GRP78 also functioned as a receptor of astrocyte-secreted NLK (Tanie et al., 2018). NLK secreted from astrocytes contributed to axonal growth in cultured neurons. Therefore, NLK-GRP78 signaling may be a beneficial signaling that leads to axonal growth directly in neurons and astrocytes and may be considered as an effective therapeutic signaling for SCI.

AMFR is a NLK receptor expressed in neurons (Leclerc et al., 2000). Several reports have shown that NLK activates the MAPK pathway *via* AMFR, leading to resistance to anticancer agents and the production of matrix metalloproteinases in tumor cell lines (Haga et al., 2008; Kho et al., 2013). Here, we did not observe an inhibitory effect of the neutralizing antibody against AMFR on NLK-induced axonal growth, indicating that AMFR does not participate in the axonal growth activity of NLK.

In addition to AMFR, other NLK receptors have been reported to be expressed in tumor cell lines. The binding of NLK to HER2 induces HER2 phosphorylation, metalloprotease-mediated ectodomain shedding, and the activation of PI3K and MAPK signaling, resulting in the ablation of the inhibitory effect of trastuzumab on cell growth in breast cancer cells (Kho et al., 2013). The interaction of NLK with G protein coupled estrogen receptor 1 (GPER-1) contributes to cell growth by activating the PI3K signaling in endometrial cancer cells (Li et al., 2019). Several studies reported the expression of GPER-1 (Gingerich et al., 2010; Srivastava and Evans, 2013; Zhao et al., 2016) but not HER2 in neurons. Although we could not exclude completely involvement of GPER-1 in NLK signaling in neurons, the neuronal cell surface-expressed GRP78 may be the main receptor involved in NLK-induced functional recovery of SCI. This is strongly supported by our data showing that the neutralizing antibody against GRP78 completely diminished the effects of NLK *in vitro* and *in vivo*.

Our study revealed NLK-GRP78 signaling as a new mechanism promoting axonal growth mediated by cell surface GRP78 in neurons, suggesting that it may play an important role in the recovery of motor function in SCI. Potential candidates of therapeutic medicine for SCI include not only NLK itself, but also other synthesized ligands to cell surface receptor GRP78. Translational research resulting in practical clinical application is likely to occur in the future.

## Data Availability Statement

All datasets generated for this study are included in the article/supplementary material.

## Ethics Statement

The animal study was reviewed and approved by The Committee for Animal Care and Use at the Sugitani Campus of the University of Toyama.

## Author Contributions

YT, TK, and CT designed the experiments and wrote the manuscript. YT conducted the experiments and analyzed the data. CT supervised all the experiments and analysis.

## Funding

This study was supported by KAKENHI Grant from the Japan Society for the Promotion of Science (Number JP26670044 and JP17H03558), a Grant-in-Aid for a Cooperative Research Project from the Institute of Natural Medicine, University of Toyama, in 2014 and 2015, and discretionary funds of the president of the University of Toyama, in 2014, 2015, 2016, and 2017, and the joint research program of Biosignal Research Center, Kobe University in 2016.

## Conflict of Interest

The authors declare that the research was conducted in the absence of any commercial or financial relationships that could be construed as a potential conflict of interest.
